# Conductive AFM for CNT characterization

**DOI:** 10.1186/1556-276X-8-24

**Published:** 2013-01-11

**Authors:** Marius Toader, Holger Fiedler, Sascha Hermann, Stefan E Schulz, Thomas Gessner, Michael Hietschold

**Affiliations:** 1 Solid Surfaces Analysis Group, Institute of Physics, Chemnitz University of Technology, Chemnitz 09107, Germany; 2 Center for Microtechnologies, Chemnitz University of Technology, Chemnitz 09126, Germany; 3 Fraunhofer Institute for Electronic Nano Systems, Chemnitz 09126, Germany

**Keywords:** Multi-walled carbon nanotubes, CNT, Conductive AFM, Atomic force microscopy, Interconnect system

## Abstract

We report on and emphasize the versatility of conductive atomic force microscopy in characterizing vertically aligned carbon nanotubes (CNTs) aimed to be used in via interconnect technology. The study is conducted on multi-walled CNT arrays vertically grown on a copper-based metal line. Voltage-dependent current mapping and current–voltage characteristics recorded down to single CNT allow for a comprehensive insight into the electric behaviour of the hybrid structure.

## Background

Starting with the pioneering work of Iijima [1], it is undeniable nowadays that the outstanding properties of carbon nanotubes (CNTs) recommend them for a wide range of applications as emphasized in a review article by Avouris et al. [2]. There is already more than one decade since the first successful report on CNT’s suitability for field-effect transistor applications [3] which started enormous aspiration toward CNT-based hybrid (nano)electronics. However, the development of a novel hybrid technology is a slow process full of challenges. The most considerable drawback represents controlling and optimizing the quality of the CNT/metal contact. Especially, in vertical interconnect systems, this issue becomes of crucial importance. However, intense work was carried out in the last decade [4-10] to develop such CNT-based hybrid interconnect systems since outstanding properties like ballistic transport [11], high thermal conductivity [12,13] and capability to carry high current densities [7,14] earn them superiority over the traditional metals used currently in the interconnect industry. For example, current densities up to 10^9^ A cm^−2^[14] and a thermal conductivity of 3,500 W m^−1^K^−1^[13] are 3 and 2 orders of magnitude superior to copper, respectively. Consequently, advanced hybrid interconnects where copper will be replaced by CNTs will represent the breakthrough in overcoming the actual limitations of the interconnect industry strongly constrained by the shrinking issues. However, the quantum resistance of CNTs requires for highly dense arrays integrated in parallel. Vertically grown CNTs using a bottom-up approach will fulfil this request and furthermore increase the possibility of achieving narrower vias with higher aspect ratio. To successfully proceed with the development and improvement of such systems, a comprehensive understanding is required and therefore a detailed characterization should be addressed. This is not an easy task since the downscaling tendency will require a characterization down to nanoscale where big challenges like confinement can occur. As a result, effects confined down to nanoscale can play a major and defining role in the overall performance of future devices. Therefore, not only the access of nanoscale is strongly required, but also the corresponding understanding is a key factor for reaching a success. Addressing these two aspects, the scanning probe microscopy techniques exhibit strong versatility. In particular, for interconnect systems, the electric characterization which gives an insight into the CNT/bottom line contact quality is of great importance. Multi-walled CNT (MWCNT)-based via interconnect systems are mainly characterized in the literature using classical electrical measurements where the entire via is contacted using a top metal electrode. It is obvious where the problem lies in this configuration. The outcome of the study tells nothing about fluctuations inside the via itself. The interpretation of such results is rather blind relative to a possible inhomogeneous internal performance. Via a (nano)characterization of such systems by conductive atomic force microscopy (c-AFM), this issue is not overlooked. Moreover, c-AFM gives the opportunity to address single CNTs earning undeniable superiority over the classical electrical measurements. While general information can be collected over an extended CNT array using the so-called current mapping, individual CNTs can be addressed in detail using current–voltage (*IV*) studies. The facility is crucial as the downscaling tendency boosts the importance of the CNT/metal contacts in the ultimate nanoscaled devices with a strong impact over the final performance. Therefore, c-AFM was applied in this work to address the electric characterization of vertically aligned MWCNT arrays grown on a copper-based metal line.

## Methods

Vertically aligned MWCNT arrays were grown by chemical vapour deposition on a copper-based conductive metal line as comprehensively described in [8,15]. The copper-based metal line is a layer stack where Ta was used as the top layer. Moreover, TaN was used as the barrier layer to inhibit copper diffusion into the Ni catalyst layer. It was shown that the lack of such a diffusion barrier would strongly affect the quality of the CNT vertical growth [8]. All data shown within this work were recorded under ambient conditions using a 5500 AFM from Agilent Technologies (Santa Clara, CA, USA). N-type (phosphorus-doped) silicon-etched AFM probes from MikroMasch (Wetzlar, Germany) with a nominal uncoated tip radius of 10 nm were used for tapping-mode imaging. In contact-mode imaging, conductive AFM probes are required in order to simultaneously obtain the corresponding current map. The Ti-Pt coating material consists of a 10-nm Pt layer on top of a 20-nm Ti sublayer and is formed on both tip and reflective side of the cantilever, leading to a nominal tip radius of around 40 nm. In the conductive AFM setup, a special nose cone with a built-in preamplifier is used for current detection when a bias voltage is applied between the sample and the cantilever. The two-terminal setup uses the conductive AFM probe as the first electrode (which contacts the top end of the MWCNTs) and a metallic wire as the second electrode (which contacts the bottom metal line via a large area of MWCNTs covered with silver paste). Every *IV* set shown within this work is, on average, over ten spectra recorded in the same contact point. One hundred points within the indicated voltage range and 2-s acquisition time were used for individual spectrum.

## Results and discussion

**Figure 1:**
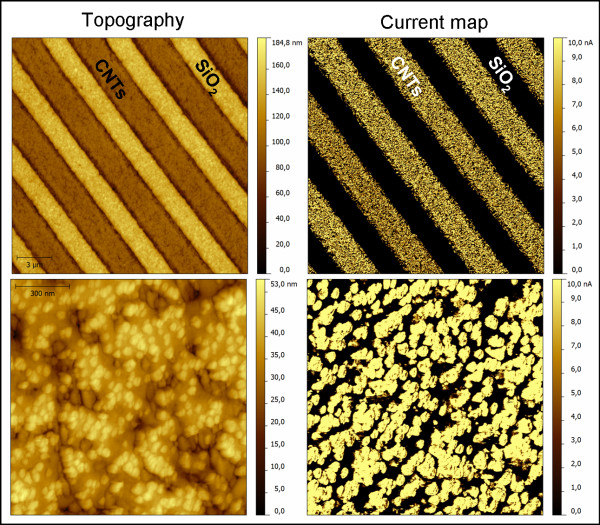
Topography (left column) vs. current map (right column).

Classical topography vs. current map AFM images are displayed in Figure 1. They can be simultaneously recorded in c-AFM configuration operating in contact mode. Trench-like CNT arrays are separated via SiO_2_ as marked in Figure 1. When a sample bias of 500 mV is applied, a current flow is generated between the bottom metal line and the metallic tip via the vertically aligned MWCNTs. While a strong signal from the CNT arrays can be identified in the current map, there is no current detected at the SiO_2_ side. At a first view, the system seems to exhibit a perfect homogeneous conductivity within the MWCNT arrays. However, the observation is misleading since the measured current exceeds the maximum 10 nA detectable with our system.

Therefore, the current map is recorded within the saturation regime which can be avoided using much lower sample biases as it will be shown later on. However, at this point, it is sufficient to emphasize a successful electric connectivity of the CNTs to the bottom metal line. High resolution down to single MWCNT is accessible via AFM. The corresponding electric response can be addressed as well, which earns AFM superiority over the classical electric measurements where the entire MWCNT array is contacted using top electrodes. Determining the CNT density and taking into consideration the AFM tip radius, it was obtained that the AFM tip gets in contact with (1.1 ± 0.1) CNTs [15]. What can be seen in the highly resolved AFM image is only the top end of the MWCNTs. The CNTs are well embedded in a SiO_2_ matrix to ensure stabilization during chemical–mechanical planarization. It can be observed from the corresponding current map that the current flows exclusively at the CNT site and drops immediately to zero at the SiO_2_ site, indicating the lack of lateral leakage currents.

The lateral resolution is well known to be tip-convoluted, and therefore, a reliable CNT diameter estimation is not possible from these measurements. Especially, in c-AFM where the AFM tip is rastering in contact mode at the sample surface, the tip convolution is greater since the metal coating leads to an enlarged tip radius. There exist some reports where this issue is carefully addressed and solutions are proposed.

**Figure 2:**
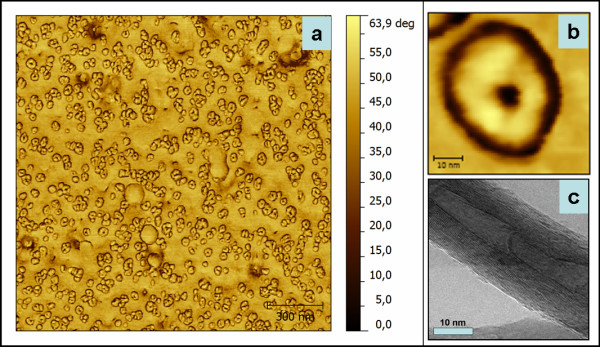
**High-resolution AFM phase images and TEM image of MWCNT.** High-resolution AFM phase images inside the MWCNT array (**a**) and of a single MWCNT (**b**); TEM image of a single MWCNT (**c**).

For example, in lying CNTs, the tip diameter estimation is done according to the height appearance which however was shown to become problematic for larger diameters due to the tip-induced deformation which results into a non-circular cross section of the CNT [16]. To reduce the tip convolution and to further increase the lateral resolution in c-AFM down to 1 nm, Hong et al. [17] have manufactured an atomic-size metallic filament on a commercial AFM probe. In our case, using the conventional tapping mode, the tip convolution can be considerably reduced. Here, uncoated pure silicon tips allow for recording high-resolution AFM images with much better improved lateral resolution. Furthermore, phase imaging provides a better contrast where the edges of individual CNTs can be distinguished more easily. The top end of individual CNTs appears as a disc-like shape with a shallow depression in the middle (see Figure 2a). According to the grain size statistics, a mean value of 20 nm was obtained with a filling percentage of 43%. A highly resolved AFM phase image of an individual CNT is displayed in Figure 2b. A corresponding transmission electron microscopy (TEM) image of a single MWCNT grown under the same conditions is shown in Figure 2c. There can be observed a very good agreement between the AFM and TEM images concerning the tube diameter.

**Figure 3:**
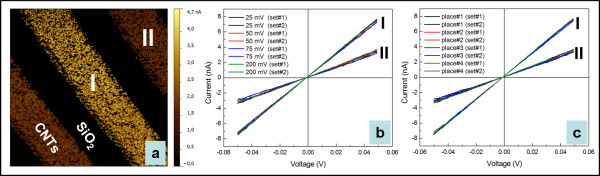
**Current map and the corresponding *I*-*V *characteristics.** Current map (**a**); the corresponding *I*-*V* characteristics for the indicated MWCNT arrays in (a) recorded under different initial sample voltages (**b**) on different locations (**c**).

**Figure 4:**
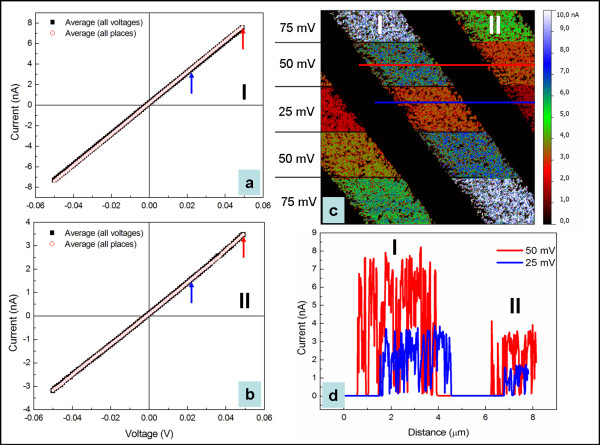
**Average *I*-*V *characteristics of MWCNT arrays, voltage-dependent current map and corresponding profile lines.** The average *I*-*V* characteristics of the MWCNT arrays I (**a**) and II (**b**); the voltage-dependent current map (**c**); the corresponding profile lines (**d**).

If the current map is recorded using a much lower sample bias of only 25 mV, variations in the electric response between distinct CNT arrays can be observed despite the good inside homogeneity (see Figure 3a). A detailed insight into the electric behaviour can be addressed by *I*-*V* spectroscopy. Here, two types of experiments were performed. On one hand, different initial sample biases were used to check if there is any influence on the *I*-*V* spectroscopy of presumably different initial loading forces induced by slight variations in the electric field between the metallic tip and the MWCNTs expected to be metallic. On the other hand, *I*-*V* spectroscopy was performed on distinct locations to get an insight into the MWCNT array homogeneity. The average spectra for the selected MWCNT arrays I and II are displayed in Figure 4a,b, respectively.

The outcome proves that none of both experiments influences somehow the electric response and sustains a very good reproducibility of the *IV* spectroscopy. The estimated average error bar approaches 2% and 4% relative to the average resistance determined for the selected I and II MWCNT arrays, respectively. Similar conductivity obtained on distinct locations supports the current mapping in what concerns the good homogeneity inside individual MWCNT arrays. The obtained linear *IV* spectra indicate that the metallic character of the MWCNTs is in good agreement with the results obtained from Raman spectroscopy and TEM studies [8]. It is more important to highlight that the formation of the MWCNT/metal contact preserves the metallic behaviour which however is not always necessarily the case. Furthermore, voltage-dependent current mapping allows probing the electric response upon a couple of sample biases at one glance (see Figure 4c). This type of study is mostly recommended and helpful for very small objects like, for example, lying CNTs, where the tip positioning and consequently a reproducible tip-CNT contact geometry becomes problematic. However, in this case, it can be furthermore used to check the correlation with the *IV* spectroscopy. In Figure 4d, two profile lines are depicted for two different sample biases, namely 50 mV (red line) and 25 mV (blue line) (refer to Figure 4c as well). The pointing-up arrows (refer to Figure 4a,b) obeying the same colour code indicate the current values obtained via *IV* spectroscopy for the previously mentioned sample biases. A very good agreement between the *IV* spectroscopy and the voltage-dependent current mapping can be clearly observed. The outcome looks very promising in investigating long and narrow nano-objects. As, for example, a lying single-walled CNT (with a length in the micron range but a diameter of only 1 nm) can presumably be very accurately sectioned via the voltage-dependent current mapping rather than performing uncertain *IV* spectroscopy with random tip-CNT contact geometry. The few obtained *IV* points will be sufficient to get a trend and therefore an insight into the electric behaviour (linear or non-linear).

**Figure 5:**
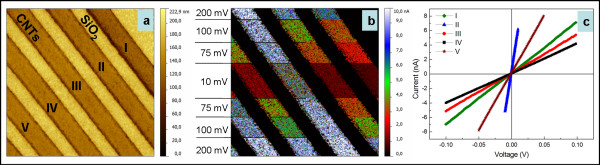
**Topography (a) vs. voltage-dependent current map (b); corresponding *I*-*V *characteristics of indicated MWCNT arrays (c).**

A similar study can be successfully extended at larger scale as can be observed from Figure 5. The same good analogy can be made between the voltage-dependent current mapping and the *I*-*V* spectroscopy. In both cases, variations in the electric response could be emphasized from array to array.

The estimated resistances of the investigated MWCNT arrays are included in Table 1. As shown previously, an error bar up to 4% might occur. Despite the relative high magnitude, the resistance values will however be considerably reduced (some orders of magnitude) for the overall highly dense vias where vertically aligned CNTs will be integrated in parallel. For example, using a rough estimation, within a 2-μm-diameter via, there can be ideally integrated (100% filling percentage) up to 10,000 MWCNTs with a diameter of 20 nm. However, if a similar filling percentage can be assumed as the one previously estimated, a correction factor of slightly larger than 2 should be included. Still, a reduced resistance of up to 3 orders of magnitude is expected to characterize the entire via. In our setup, it must be mentioned that the estimated resistances contain, besides the internal CNT resistance, inputs from metal contacts, namely metallic tip/CNT and CNT/bottom metal line. Whilst the first-mentioned top contact resistance is constant (due to the same loading force) and the CNT quality is presumably the same (Raman spectroscopy confirmed this issue on a similar sample [15]), the observed variation in the electric response from network to network is due to the bottom contact resistance. At the moment, it can be concluded that the electric behaviour of the bottom contact layer is inhomogeneous. The reason behind is mostly due to the formation of tantalum oxide and tantalum carbides as could be emphasized by energy-filtered TEM [15] which however requires for ultimate sample damage. In this regard, it was shown that c-AFM gives the extra possibility to electrically investigate buried interfaces to a very low scale being superior in this regard to the standard *IV* measurements which exhibit a strong average character.

**Table 1:** The estimated resistance values of the indicated MWCNT arrays

**MWCNT array**	**I**	**II**	**III**	**IV**	**V**
Resistance (MΩ)	24.49	19.04	1.74	14.20	6.33

## Conclusions

The final message of this work emphasizes the versatility of c-AFM to investigate vertically aligned MWCNT arrays aimed for via interconnect systems in a highly reproducible manner. Such studies can bring in parallel to the 3D topography substantial advantages over the standard *I*-*V* measurements. Complementary information confined down to extremely low scales is accessible. This might be of great relevance for future studies on extremely narrow CNTs via interconnects where the importance of individual CNTs grows considerably, especially possible variations in the electric behaviour from individual CNTs can occur. Complementary to the classical electric measurements where top contacts are required and therefore a general electric behaviour for the whole via is obtained, c-AFM can address individual CNTs and get a better detailed insight into the via. The outcome can prove itself of crucial importance in comprehensively understanding and consequently optimizing the development of via interconnect systems.

## Competing interests

The authors declare that they have no competing interests.

## Authors’ contributions

MT performed all the AFM measurements and wrote the manuscript. HF and SH developed the technology behind the sample preparation and consequently prepared the samples. Corrections to the manuscript were also provided. SS, TG and MH put the basis of the entire project, guided the internal collaboration, and read and improved the manuscript. All authors read and approved the final manuscript.
